# Potential mosquito repellent compounds of *Ocimum* species against 3N7H and 3Q8I of *Anophelesgambiae*

**DOI:** 10.1007/s13205-015-0346-x

**Published:** 2016-01-11

**Authors:** Venugopal Gaddaguti, Talluri Venkateswara Rao, Allu Prasada Rao

**Affiliations:** 1 Department of Biotechnology, KL University, Vaddeswaram, Guntur, AP 522 502 India; 2 Department of Horticulture, Sikkim University, Samdur, Tadong, Gangtok, Sikkim 737 102 India

**Keywords:** Mosquito repellent compounds, Schrodinger maestro, Molecular docking, ADME properties

## Abstract

Mosquitoes are exceptionally efficient in detecting their hosts for blood meal using odorant binding proteins, viz. 3N7H and 3Q8I and spread several dreadful diseases. DEET is a synthetic mosquito repellent widely used all over world for protection against mosquito bite. Reports reveal that, synthetic mosquito repellents may pose health problems in considerably large population. In view of the above fact, we made an attempt to discover efficient and novel natural mosquito repellent compounds with least impact on human health. Methanolic leaf extracts of *Ocimum basilicum* Linn. var. *pilosum* (*willd.*)-*Benth* and *Ocimum tenuiflorum* var. CIM-AYU were subjected to GC–MS analysis and obtained 35 phytochemical constituents. Repellent potentiality of the *Ocimum* compounds was assessed against 3Q8I and 3N7H of *Anopheles* *gambiae.* PDB structures of mosquito odorant binding proteins were downloaded, processed and docking studies were performed along with reference ligand DEET using Schrodinger MAESTRO 9.2 software. Molecular docking results reveal that phenol, 2-methoxy-3-(2-propenyl)-, licopersin, gamma sitosterol and benzene, 1,2-dimethoxy-4-(2-propenyl)- from *O. tenuiflorum* var. CIM-AYU are strongly bound with 3N7H. Whereas, 4h-1-benzopyran-4-one, 5-hydroxy-6,7-dimethoxy-2-(4-methoxyphenyl)-, catechol and monoacetin from *O. basilicum* Linn. var. *pilosum* (*willd.*)-*Benth.* show high binding affinity with odorant binding protein 3Q8I. All natural compounds tested in the present study display better docking scores than DEET. The results further substantiate that the 12 out of 35 compounds of the two *Ocimum* species found to be ideal candidates for design and development of potential mosquito repellents. ADME properties of the tested compounds further confirm that bioactive compounds of *Ocimum* species were found to be in acceptable range. Synchronized application of at least two different natural compounds (with best docking scores) which target 3N7H and 3Q8I (Odorant Binding Proteins of mosquito) proteins may provide enhanced protection against mosquitoes bite. Based on the ADME properties, natural compounds of *Ocimum* species can be considered for design and development of safe mosquito repellents.

**Electronic supplementary material:**

The online version of this article (doi:10.1007/s13205-015-0346-x) contains supplementary material, which is available to authorized users.

## Introduction

Mosquitoes act as vector for many protozoans, bacterial and viral diseases (Service 1996). Increase in mosquito population is a perennial problem for many developing countries due to spread of diseases like malaria, filarial, encephalitis, etc. To protect humans against mosquito born diseases, mosquito repellents’ in different forms (herbs, aromatic oils and synthetic compounds) were widely used all over world. Of many available synthetic mosquito repellents, the best-known chemical insect repellent is *N,N*-diethyl-3-methyl benzamide (DEET) (Fradin and Day 2002). It will be more surprising to know that over 40 years of its discovery and usage, DEET gain remarkable safety profile (Fradin 1998) with highest biting inhibition rate (88.7–92.5 %) against wide range of mosquitoes. However, studies reveal that the use of synthetic mosquito repellents at faster pace could cause many side effects (slurred speech, muscle twisting, seizures, rashes, vomiting and nausea) and elevate issues concerning human health. To safeguard human health, use of safe natural compounds from plant extracts became an alternative approach to minimize the side effects as compared to synthetic mosquito repellents. Historically it is well known that plant extracts were extensively used as potential natural repellents against wide range of insects over 2000 years ago (Nentwing 2003). Moreover, many natural herbs have been evaluated for their flavouring characters and therapeutic properties (Singh et al. 2010). However, the newly discovered natural compounds from potential medicinal plants have not been fully explored due to their toxic characteristics (Metacalf 1962).

Studies confirm that plant extracts from *Phytolacca dodencandra* showed larvicidal activity against mosquitoes (Dahlman and Hibb 1967). Since ages, ancient people use herbal plants in different ways to repel mosquitoes. Widely used practices include generation of smoke by burning plants (Sharma and Ansari 1993), hanging fresh plants in houses (Waka et al. 2006) for avoiding mosquitoes in the near vicinity. In contrast to whole herbs, studies confirm that plant extracts form wide range of plants are more advantageous and efficacious. Among the plant families with promising essential oils used as repellents, *Cymbopogon*, *Ocimum* and *Eucalyptus* are the most cited. The prime objective of the present investigation is to explore the possibility of repellent activity of potential natural compounds from *Ocimum* species against odorant binding proteins of *Anopheles gambiae.* Attempts were made to understand molecular mechanisms underlying possible interactions of natural mosquito repellent compounds against odorant binding proteins (3Q8I and 3N7H) of *A. gambiae.* Further these studies will widen the scope to choose the most suitable compounds for design and development of effective and safe mosquito repellents.

## Materials and methods

### Plant material, sample extraction, GC MS analysis and Identification of components

*Ocimum tenuiflorum* var. CIM AYU and *O. basilicum* Linn. var. *pilosum* (*willd*)-*Benth.* Seeds were sown at K L University botanical garden. Fresh and healthy leaves were powdered, extracted in methanol, concentrated and finally the active components were analysed using GC–MS (Manorenjitha et al. 2013). Interpretation of mass spectrum of GC–MS was done using the databases of National Institute Standard and Technology version (NIST08s) WILEY8, FAME.

### Molecular docking

The 2D structures of 35 compounds of two *Ocimum* species were downloaded using Chem Spider. These structures were converted into 3D form and Schrodinger-aided drug design software and used for molecular docking analysis. The 3D structures of 3N7H and 3Q8I proteins were downloaded from the RCSB (PDB) and modified to make the protein biologically active and stable. Later Protein Preparation Wizard tool of Schrödinger’s software converts a raw PDB structure into all-atom fully prepared protein. In the modified protein, the active site was identified and further optimized by removing free water molecules and hetero atoms. For docking studies, only the ideal active site was selected. For favorable interactions between one or more ligand molecules on a receptor, receptor region with optimal binding affinity grid was generated for glide searches. Ligands obtained in all aspects follow Lipinski’s rule of 5. Structures obtained from Pubchem/Chemspider database were sketched using Chem sketch tool and finally the high quality all atom 3D structures were saved in Maestro (.mol) format. Optimization of structures were performed using LigPrep tool by removing unwanted molecules, addition of hydrogen, and ligand structure minimization. Finally, Grid-based Ligand Docking with Energetics (GLIDE) was performed.

### ADME properties

Pharmacokinetic and pharmacological properties (ADME) of potential mosquito repellent ligands were studied using QikProp module of the Schrödinger 9.2 software. ADME properties predicted for the potential compounds of *Ocimum* species were programmed in accordance with Lipinski’s rule of five. On the other hand, QikProp tool is used to evaluate the bioavailability of the lead molecules by assessing their physicochemical properties to observe the range of the Lipinski rule for induced molecules (Bhogireddy et al. 2013). The chemical behaviour of the *Ocimum* compounds were evaluated through analysis of pharmacological parameters required for absorption, distribution, metabolism and excretion (ADME) and Rule of five and Rule of three (QikProp and Version 2012).

## Results and discussion

The qualitative analysis of components from methanolic leaf extracts of *O. tenuiflorum* var. *CIM AYU* (20) and *Ocimum basilicum* Linn. var. *pilosum* (*willd*)-*Benth.* (15) yielded total of 35 compounds. Of the 35 compounds tested for their binding affinity with odorant binding proteins of *Anopheles* mosquito, 2-hexadecen-1-ol, phytol, dl-alpha-tocopherol, phenol-2-methoxy-3-(2-propenyl)-, lycopersin, gamma-sitosterol, benzene, 1,2-dimethoxy-4-(2-propenyl)- of *O. tenuiflorum* exhibit strong binding affinity with odorant binding protein 3N7H. Whereas, 4H-1-benzopyran-4-one, 5-hydroxy-6,7-dimethoxy-2-(4-methoxyphenyl), catechol, phytol, 2-hydroxy-6-methylbenzaldehyde, Monoacetin from *O. basilicum* Linn. var. *pilosum* (*willd*)-*Benth* display intense binding affinity with 3Q8I of *A. gambiae* (Table 1).

**Table 1:** Potential compounds of *O. tenuiflorum* var. CIM-AYU *and O. basilicum* Linn. var. *pilosum* (*willd*)-*Benth*

S. No	Name of the plant species	Name of the compound
1	*O. basilicum* Linn. var. *pilosum* (*willd*)-*Benth.*	4H-1-Benzopyran-4-One, 5-Hydroxy-6,7-Dimethoxy-2-(4-Methoxyphenyl) Catechol Phytol 2-Hydroxy-6-methylbenzaldehyde Monoacetin
2	*Ocimum tenuiflorum* var. CIM AYU	2-Hexadecen-1-ol Phytol dl-alpha-Tocopherol Phenol-2-Methoxy-3-(2-Propenyl)- Lycopersin Gamma-Sitosterol Benzene, 1,2-Dimethoxy-4-(2-Propenyl)-

The G scores, H bonding with corresponding amino acids of potential compounds of two *Ocimum* species against mosquito odorant binding proteins (3Q8I and 3N7H) along with reference ligands are presented in Table 2. Particularly, DEET demonstrated a strong affinity with 3Q8I receptor and forms hydrogen bonding (−0.7) and selectively bound to THR 57 amino acid residue with a glide score of −5.13. Similarly, 4 h-1-benzopyran-4-one, 5-hydroxy-6,7-dimethoxy-2-(4-methoxyphenyl) and catechol, exhibit affinity with THR 57 amino acid residue and both compounds characteristically exhibit hydrogen bonding. The receptor pocket of 4h-1-benzopyran-4-one, 5-hydroxy-6,7-dimethoxy-2-(4-methoxyphenyl)- is enclosed with hydrophobic and polar region. Whereas, the receptor pocket of Catechol is completely polar in nature. However, former exhibits side chain hydrogen bonding and later displays backbone hydrogen bonding. The glide scores recorded for the aforesaid compounds were −7.14 and −6.49, respectively. On the other hand, the two compounds namely, Phytol, and Monoacetin shown to bind with more than one amino acid residues. Phytol typically forms backbone hydrogen bonding with two hydrophobic amino acid residues namely MET 122 and PHE 121 with glide scores of −6.42 and −5.56, respectively. Monoacetin exhibits hydrogen bonding with a hydrophobic (ALA 106) and polar (THR 57) amino acid residues. Both ligands establish backbone hydrogen bonding with their respective amino acids. Of the five compounds tested from *O. basilicum* Linn. var. *pilosum* (*willd*)-*Benth.,* 2-hydroxy-6-methylbenzaldehyde exclusively bound to hydrophobic amino acid residue ALA 106 with backbone hydrogen bond with a glide score of −5.56. Moreover, this ligand exhibits additional pi–pi stacking interaction with PHE 123. All five compounds of *O. basilicum* show hydrogen bonding (supplementary Fig. 1a) and found to exhibit relatively higher G scores than reference ligand DEET (Table 2). Moreover, formation of hydrogen bonding with receptor 3Q8I further strengthens the possibility that these compounds would serve as potential mosquito repellents.

**Table 2:** Ligands showing good affinity with the receptors 3Q8I and 3N7H

Ligand	G-score	H-bond	Residue
3Q8I protein			
DEET	−5.13	−0.70	THR 57
4h-1-Benzopyran-4-one, 5-hydroxy-6,7-dimethoxy-2-(4-methoxyphenyl)-	−7.14	−1.86	THR 57
Catechol	−6.49	−3.02	THR 57
Phytol	−6.42	−1.25	MET 122, PHE 121
2-Hydroxy-6-methylbenzaldehyde	−5.56	−1.56	ALA 106
Monoacetin	−5.45	−3.25	THR 57, ALA 106,
3N7H protein			
DEET	−2.74	−0.63	ASN 56
2-Hexadecen-1-ol	−4.98	−0.35	GLU 49
Phytol	−4.64	−0.35	GLU 49
dl-alpha-tocopherol	−4.54	−0.35	GLU 49
Phenol-2-methoxy-3-(2-propenyl)-	−4.03	−1.92	ASN 56, CYS 53
Lycopersin	−4.01	−1.45	ASN 56, LYS 29
Gamma-sitosterol	−3.86	−1.13	ASN 56
Benzene, 1,2-dimethoxy-4-(2 propenyl)-	−3.11	−0.68	ASN 56

Unlike 3Q8I, the odorant binding receptor protein of *A. gambiae* (3N7H) exhibit differential binding pattern with the compounds of *O. tenuiflorum* var. CIM AYU. Studies further reveal that the reference ligand DEET has affinity with 3N7H receptor protein and forms hydrogen bonding (−0.63) and selectively bound to ASN 56 amino acid residue with a glide score of −2.74. Of the seven compounds tested as against 3N7H, 2-hexadecen-1-ol, phytol and dl-alpha-tocopherol shown to have backbone hydrogen bonding by negatively charged GLU 49 amino acid residue with glide scores of −4.98, −4.64 and −4.54, respectively. Further the receptor pockets of these ligands were surrounded by hydrophilic and hydrophobic regions.

Similarly, gamma-sitosterol and benzene, 1,2-dimethoxy-4-(2 propenyl)- were found to interact with polar amino acid residue ASN 56 and recorded glide scores of −3.86 and −3.11, respectively. The ligand gamma-sitosterol establishes backbone hydrogen bonding and the receptor pocket is surrounded by hydrophobic region. Whereas benzene, 1,2-dimethoxy-4-(2 propenyl)- forms side chain hydrogen bonding and the pocket is enclosed by hydrophobic and hydrophilic areas correspondingly. Whereas phenol-2-methoxy-3-(2-propenyl)- and lycopersin also bound to ASN 56 amino acid residue but the former share with CYS 53 and the latter with LYS 29 amino acid residue. In contrast, ligand phenol-2-methoxy-3-(2-propenyl)- receptor pocket is totally surrounded by hydrophobic region. Interestingly, this ligand establishes backbone hydrogen bonding with hydrophobic (CYS 53) and Polar (ASN 56) amino acid residues respectively (Supplementary Fig. 1b). Moreover, all compounds tested in the present study exhibit hydrogen bonding and also display hydrophobic activity with corresponding amino acids. Calculated ADME properties for all potential compounds of two *Ocimum species* were found to be in the normal range (Table 3).

**Table 3:** ADME properties of potential compounds of *O. tenuiflorum* var. CIM-AYU and *O. basilicum* Linn. var. *pilosum* (*willd*)-*Benth. Ocimum*

Plant/variety	Ligands	MW	QP log Kp	Donor HB	Accpt HB	R of F	R of T
Normal range		130–725	−8.0 to 1.0	0–6	2–20	Max 4	Max 3
*Ocimum tenuiflorum* var. CIM-AYU	2-Hexadecen-1-ol	256.22	−1.18	0	2.0	0	0
	Phytol	256.22	−1.18	0	2.0	0	0
	dl-alpha-Tocopherol	380.32	−1.55	0	4.0	0	0
	Phenol-2-Methoxy-3-(2-Propenyl)-	152.11	−2.50	0	2.5	0	0
	Lycopersin	368.22	−3.97	0	11.5	0	0
	Gamma-Sitosterol	364.32	−1.73	0	2.0	0	0
	Benzene, 1,2-Dimethoxy-4-(2 Propenyl)-	164.12	−2.18	0	4.0	0	0
*O. basilicum* Linn. var. *pilosum* (*willd*)-*Benth.*	4h-1-Benzopyran-4-One, 5-Hydroxy-6,7-Dimethoxy-2-(4-Methoxyphenyl)-	328.32	−2.59	0	4.5	0	0
	Catechol	110.11	−2.51	2	1.5	0	0
	Phytol	256.22	−1.18	0	2.0	0	0
	2-Hydroxy-6-methylbenzaldehyde	136.12	−2.51	0	1.7	0	0
	Monoacetin	124.11	−4.34	0	6.5	0	0

In the context of identifying functional compounds, docking became very important tool (Schneider and Bohm 2002; Wasz Kowycz 2002; Toledo-Sherman and Che 2002) used during drug designing process. It is a process wherein two molecules fit together in 3D space. Of the 35 compounds tested in the present study from two *Ocimum* species, only 7 compounds (phenol, 2-methoxy-3-(2-propenyl)-, licopersin, gamma sitosterol, and benzene, 1,2-dimethoxy-4-(2-propenyl, 2-hexadecen-1-ol, phytol and dl-alpha-tocopherol) found potential against 3N7H with respect to glide score, H-bonding and amino acid residues. Whereas the other 5 compounds investigated against 3Q8I receptor, three compounds namely, 4h-1-benzopyran-4-one, 5-hydroxy-6,7-dimethoxy-2-(4-methoxyphenyl)-, catechol and monoacetin display higher G Score against 3Q8I when compared to phytol and 2-hydroxy-6-methylbenzaldehyde. The study further supports the earlier works of *Ocimum* plants used as mosquito repellents (Dekker et al. 2011; Koech and Mwangi 2013). Overall high binding affinity between ligand and the target protein is indicated by high negative value of glide score.

## Conclusion

In the present study, active sites of the receptor 3Q8I and 3N7H were determined and docked with the compounds obtained from GC–MS analysis from two *Ocimum* (*O. tenuiflorum* var. CIM AYU and *O. basilicum* Lin. var. *pilosum* (willd.)-Benth.] species. Of the 35 compounds tested from two *Ocimum* species, 12 compounds are properly docked with *A. gambiae* odorant binding proteins (3N7H and 3Q8I).

After careful evaluation of ADME parameters such as *MW* (Molecular weight), *QPlog Kp* (Predicted skin permeability), *donor HB* (Estimated number of hydrogen bonds that would be donated by the solute to water molecules in an aqueous solution), *accpt HB* (Estimated number of hydrogen bonds that would be accepted by the solute from water molecules in an aqueous solution), according to *Rule of Five* (Lipinski et al. 2001), all compounds satisfy these rules and were considered drug like. On the other hand, number of violations of Jorgensen’s rule of three (QPlogS (Predicted aqueous solubility), QP PCaco (Predicted apparent Caco-2 cell permeability in nm/s. Caco-2 cells are a model for the gut–blood barrier. QikProp (predictions are for non-active transport) (Jorgensen and Duffy 2002) was also tested for all 12 compounds and found that all 12 compounds have passed with fewer or no violations. Hence these compounds were more likely to be accepted for oral administration though these were not intended for oral administration. The results were checked with the reference ligand DEET. ADME properties of these bioactive compounds were in acceptable range. Therefore it can be concluded that these bioactive compounds may act as novel compounds and can be used as an efficient and safer mosquito repellent than that of commercially available DEET.

**Fig. 1:**
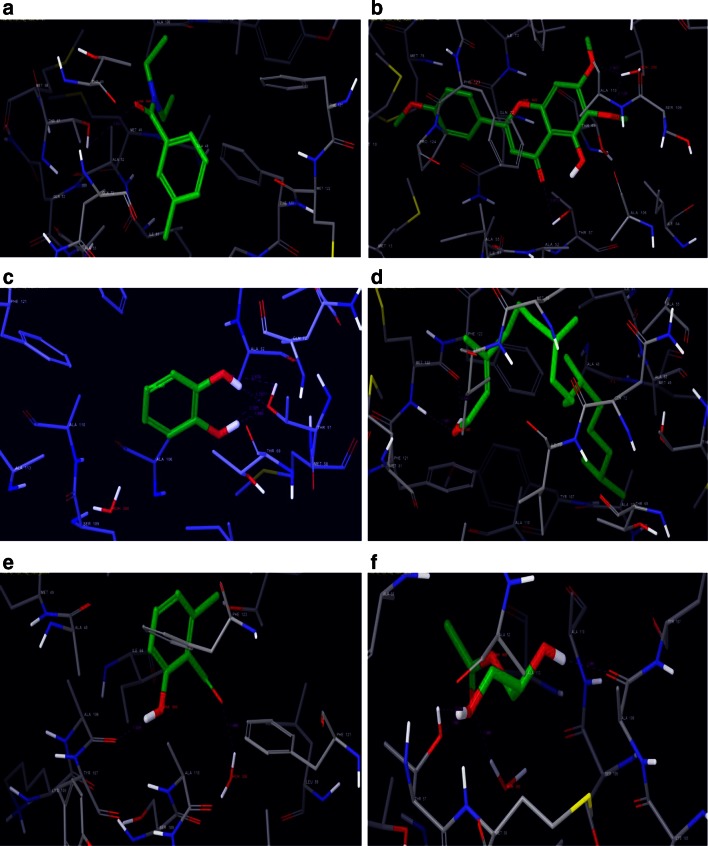
Molecular docking of *O. basilicum* Linn. var. *pilosum* (*willd*)*-Benth* with 3Q8I. **a** Reference ligand DEET with 3Q8I. **b** 4h-1-Benzopyran-4-one, 5-hydroxy-6,7-dimethoxy-2-(4-methoxyphenyl)- with 3Q8I. **c** Catechol with 3Q8I. **d** Phytol with 3Q8I. **e** 2-Hydroxy-6-methylbenzaldehyde with 3Q8I. **f** Monoacetin with 3Q8I

**Fig. 2:**
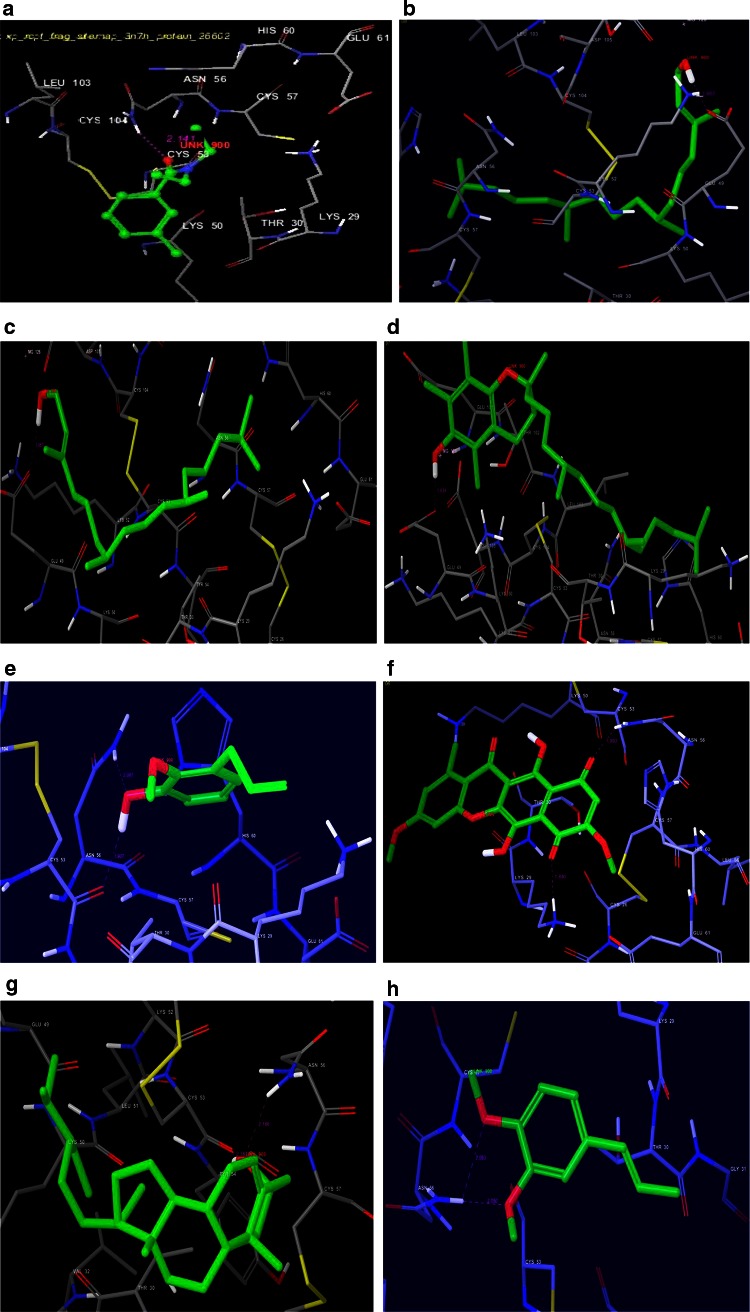
Docking 3N7H With *Ocimum tenuiflorum* var. CIM-AYU. **a** Docking 3N7H With *Ocimum tenuiflorum* var. CIM-AYU. DEET with 3N7H. **b** 2-Hexadecen-1-ol with 3N7H. **c** Phytol with 3N7H. **d** d–α-Tocopherol with 3N7H. **e** Phenol-2-methoxy-3-(2-propenyl)- with 3N7H. **f** Lycopersin with 3N7H. **g** γ-Sitosterol with 3N7H. **h** Benzene, 1,2-Dimethoxy-4-(2-propenyl)- with 3N7H

Till date 69 odorant binding proteins have been reported in *A. gambiae* and typically all OBPs may not be involved in recognition of host for their blood meal. However, the two OBPs namely 3N7H and 3Q8I at least in part play a crucial role in identification of host. The present study aimed to compare the binding affinity of natural mosquito repellent compounds of two *Ocimum* species against commercially available mosquito repellent DEET. The compounds tested in the present study seem to be potential enough to repel mosquitoes effectively by binding either 3Q8I or 3N7H of *A. gambiae* independently. These repellents interfere with the function of odour binding proteins and in turn play a similar or even better role like DEET in preventing mosquitoes from finding their prey. Moreover, simultaneous application of more than two potential mosquito repellent compounds which target multiple odorant binding proteins may provide optimum protection from mosquito bite. For integrated control of mosquitoes, further research in this direction is very much required. Validation of natural mosquito repellent compounds from novel aromatic plant species may open new avenues for design and development of eco and user friendly mosquito repellents (Figs. 1, 2).

## Electronic supplementary material

Below is the link to the electronic supplementary material.

Supplementary material 1 (DOCX 844 kb)
