# Supplementary material for: Potential mosquito repellent compounds of Ocimum species against 3N7H and 3Q8I of Anophelesgambiae
Source: 3 Biotech. 2016 Jan 11;6(1):26. doi: 10.1007/s13205-015-0346-x (PMC4711284; doi:10.1007/s13205-015-0346-x)

**3Q8I (Supplementary Figure 1 A)**


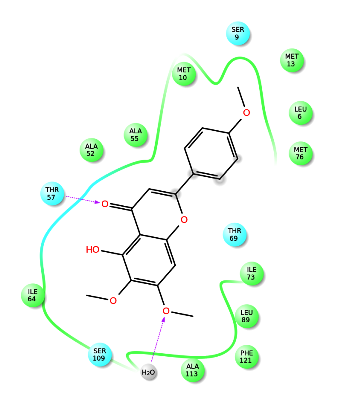

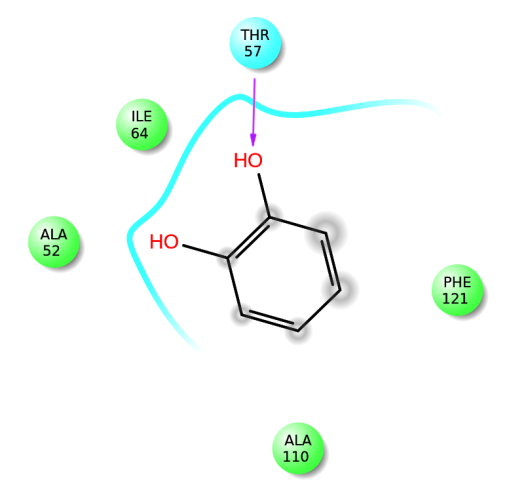

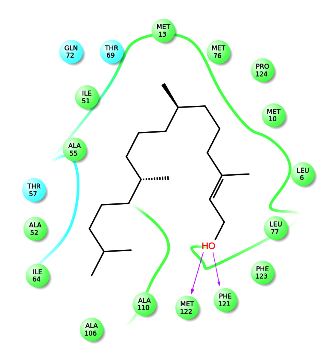


4h-1-Benzopyran-4-One, 5-Hydroxy-6, Catechol Phytol

7-Dimethoxy-2-(4-Methoxyphenyl)-


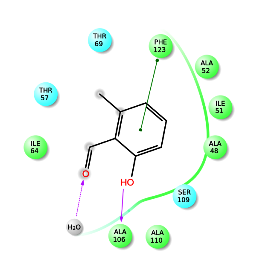

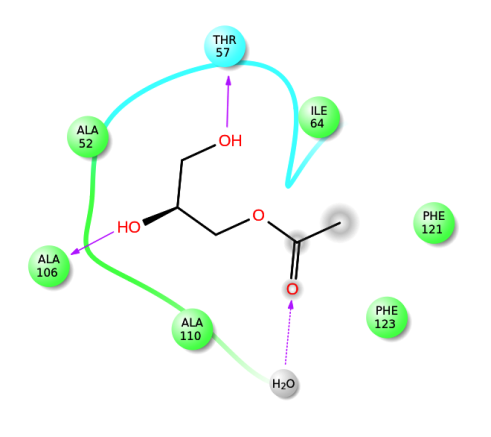


2-Hydroxy-6-methylbenzaldehyde Monoacetin

**3N7H (Supplementary Figure 1 B)**


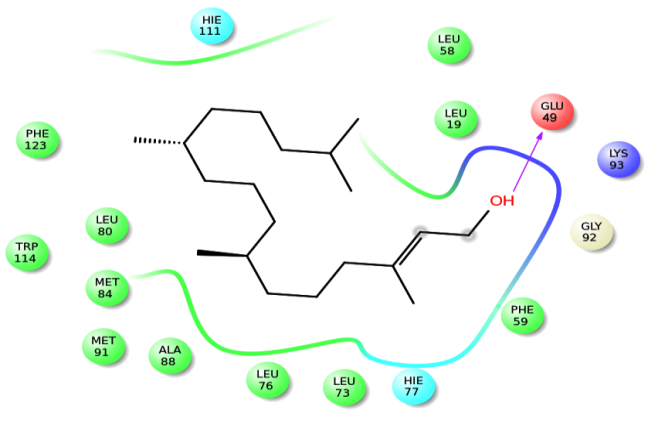

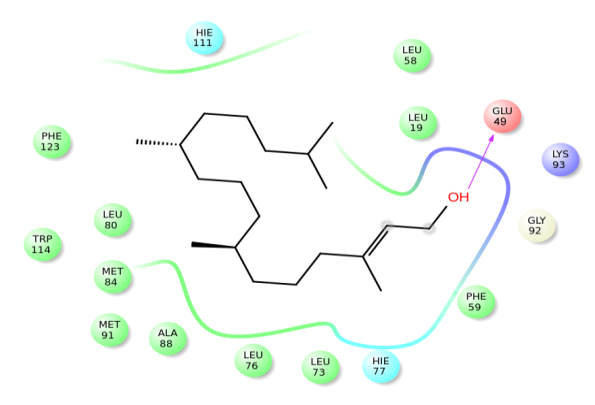

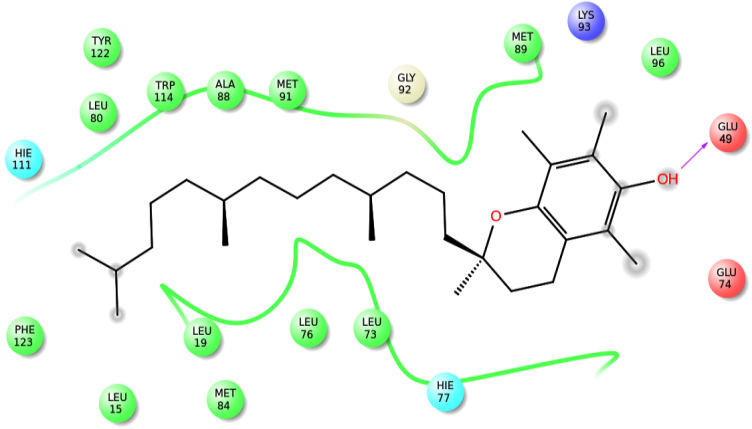


2-Hexadecen-1-ol Phytol dl-alpha-Tocopherol


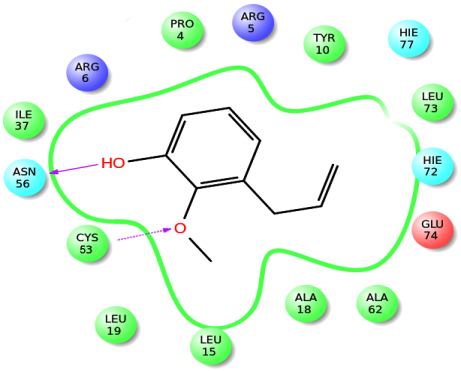

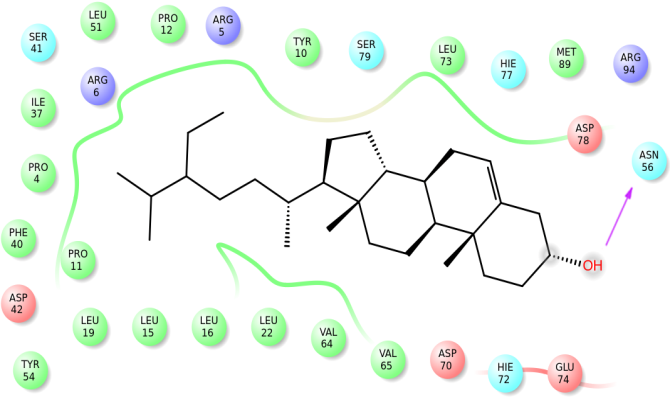

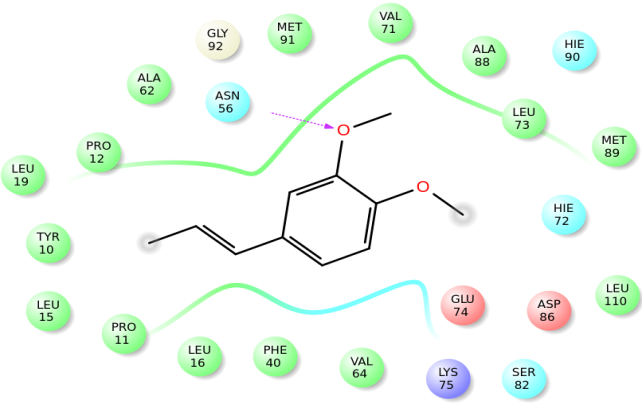


Phenol- 2-Methoxy-3-(2-Propenyl)- Gamma-sitosterol Benzene, 1,2-Dimethoxy-4-(2 Propenyl)-


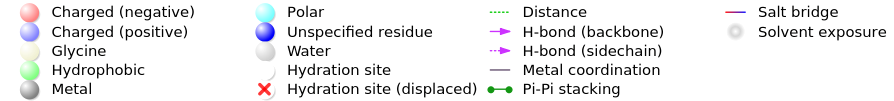

Supplement: Supplementary file 1 — Supplementary material 1 (DOCX 844 kb) [file 13205_2015_346_MOESM1_ESM.docx]
